# Multimodal Assessment of Therapeutic Alliance: A Study Using Wearable Technology

**DOI:** 10.3390/jemr18040036

**Published:** 2025-08-12

**Authors:** Mikael Rubin, Robert Hickson, Caitlyn Suen, Shreya Vaishnav

**Affiliations:** 1 Department of Psychology, Palo Alto University, Palo Alto, CA 94304, USA; rhickson@paloaltou.edu (R.H.); ksuen@paloaltou.edu (C.S.); 2 Department of Counseling, Palo Alto University, Palo Alto, CA 94304, USA; svaishnav@paloaltou.edu

**Keywords:** therapeutic working alliance, trainees, quantitative methods, eye tracking, technology

## Abstract

This empirical pilot study explored the use of wearable eye-tracking technology to gain objective insights into interpersonal interactions, particularly in healthcare provider training. Traditional methods of understanding these interactions rely on subjective observations, but wearable tech offers a more precise, multimodal approach. This multidisciplinary study integrated counseling perspectives on therapeutic alliance with an empirically motivated wearable framework informed by prior research in clinical psychology. The aims of the study were to describe the complex data that can be achieved with wearable technology and to test our primary hypothesis that the therapeutic alliance in clinical training interactions is associated with certain behaviors consistent with stronger interpersonal engagement. One key finding was that a single multimodal feature predicted discrepancies in client versus therapist working alliance ratings (b = −4.29, 95% CI [−8.12, −0.38]), suggesting clients may have perceived highly structured interactions as less personal than therapists did. Multimodal features were more strongly associated with therapist rated working alliance, whereas linguistic analysis better captured client rated working alliance. The preliminary findings support the utility of multimodal approaches to capture clinical interactions. This technology provides valuable context for developing actionable insights without burdening instructors or learners. Findings from this study will motivate data-driven methods for providing actionable feedback to clinical trainees.

## 1. Introduction

### Interpersonal Interactions

Interpersonal interactions are foundational to effective clinical practice, making the development of interpersonal skills a critical component of healthcare provider training [1]. In clinical training, practicing therapy skills prepares future healthcare professionals to navigate complex patient interactions, address diverse needs, and deliver compassionate care. Strong interpersonal skills contribute to better patient outcomes [2,3,4]. Skills such as active listening, empathy, working alliance, building trust, and collaboration have been conceptualized as “common factors”—atheoretical elements pervasive across treatment modalities and crucial to therapeutic relationships [5,6]. However, of these “common factors,” therapist–client working alliance has been identified as the most effective skill [1].

Working alliance is a tripartite model consisting of goals, tasks, and bond in a therapy session between the client and counselor [7]. It reflects the extent to which client and therapist agree on goals, identify tasks to be accomplished, and share an emotional connection. Working alliance has been shown to predict better therapy outcomes [8,9]. For example, in one study of eighty-two patients engaging in Cognitive Behavioral Therapy, stronger therapeutic alliances measured in the later stages of therapy correlated with more favorable outcomes, while weaker therapeutic relationships predicted patient dropout [9]. In a multi-level meta-analysis, Del Re et al. [8] found that the therapist’s contribution to the alliance significantly predicted treatment outcomes, even after controlling for confounding variables like research methodology, personality disorders, and outcome measures. Therefore, understanding client–therapist working alliance—especially the therapist’s contribution—can inform the skill development and training of clinicians.

Subjective assessments in healthcare education are commonly used to measure interpersonal effectiveness [10,11]; however, these measures often suffer from reliability issues and observer bias [12,13]. This highlights the need for more objective tools to evaluate interpersonal skills. Wearable technologies, particularly eye-tracking glasses, have emerged as methods for measuring attention in both task-based and naturalistic interpersonal contexts [14,15,16].

Investigating visual attention is a promising avenue for understanding interpersonal dynamics. Research indicates that attentional patterns, such as eye contact frequency and duration, correlate with clinician engagement, empathy, and active listening [17,18]. Wearable eye-tracking technology captures gaze behavior at high temporal resolution and can be integrated with other modalities like physiological and audio data [19]. Researchers have used gaze data to understand visual attention patterns [3,20]. However, findings on patient–provider interactions are mixed. For instance, one study found that more face gaze was linked to lower patient trust [21], while another found increased perceived empathy when practitioners looked at patients’ faces, especially their mouths [22]. Similarly, eye contact has been shown to coordinate speaking turns, support self-monitoring, and prevent communication breakdowns [23].

To further explore gaze-based interpersonal behavior, Cross-Recurrence Quantification Analysis (CRQA) offers a powerful, data-driven approach to assess real-time interpersonal dynamics. CRQA is a nonlinear time-series technique that quantifies temporal coordination between signals such as gaze and speech [24,25]. When applied to multimodal data, CRQA allows researchers to study how individuals align their visual and verbal behavior over time. This has important implications in healthcare education, where communication involves mutual gaze, coordinated turn-taking, and active listening [16,26]. For instance, CRQA can identify whether learners follow or lead gaze shifts in response to verbal cues, or whether they engage in reciprocal attention patterns that demonstrate empathy. Such interpersonal synchrony—of gaze and speech—has been linked to greater rapport, trust, and improved patient outcomes [25]. Integrating CRQA with wearable eye-tracking and audio capture technologies offers a nuanced and objective framework for estimating dynamic patterns in clinical engagement. These patterns can help us understand how learners engage with patients and/or peers during real or simulated clinical interactions. Leveraging wearable eye-tracking technology may lead to better understanding, training, and targeting feedback toward interpersonal skills, thus enriching the quality of healthcare education and practice.

The primary aim of this study was to test the use of wearable eye trackers (recording gaze and speech) to predict working alliance during a simulated clinical interaction. We hypothesized that therapeutic alliance in clinical training interactions would be associated with interactional dynamics based on gaze and speech pattern derived from CRQA consistent with stronger interpersonal engagement. Our second exploratory aim was to investigate client language use (i.e., the content of what the client said) as a predictor of their rating of working alliance.

## 2. Materials and Methods

### 2.1. Participants

Participants in this study were required to be 18 years or older and either enrolled in the Master’s of Clinical Mental Health Counseling program or the PhD/PsyD Clinical Psychology program at a private non-profit institution in the United States. Participants were (N = 30) graduate students completing clinical training programs on the West Coast of the U.S.A., primarily PhD students in clinical psychology (n = 23, 82%). The full demographic summary of the sample can be found in Table 1. A total of 10 triads engaged in this pilot study, but complete data was obtained from 9 triads (n = 27). Sample size was based on the funds available to the faculty to conduct the research. Participants first completed a screening survey and, if eligible, a consent form, prior to beginning the study. Total participation time was approximately 90 min, and each participant received a $60 Amazon gift card upon completion of the study as compensation. The Institutional Review Board at Palo Alto University 2023-086-PAU approved all procedures.

### 2.2. Procedures

Participants first consented to complete a screening, then completed a screening survey and self-report questionnaires to confirm their eligibility for the study and were asked “In a few sentences please describe for the research team what concern and identity you plan to bring to the roleplay as a client (please do not share this with your peers who may also participate).” Participants were eligible if they were students at the University in their 2nd year (if in the PhD program or PsyD program) or had completed the counseling skills class (if in the Masters of Clinical Mental Health Counseling program) and who were not in directly supervised by the study investigators (so that there would not be any conflicts of interest). Participants then completed the study consent. Participants were assigned to study sessions based on their schedule availability. The study sessions were organized with three participants each, with each individual alternating between therapist and client roles. The role-play pairings were structured as follows: Participant A as therapist, Participant B as client, Participant B as therapist, Participant C as client, and Participant C as therapist, Participant A as client. Participants were not informed of their pairings until they arrived at the study session. During the simulated clinical interactions, participants in the client role utilized their prepared vignettes, which were vetted for appropriateness by the researchers.

Prior to the simulated clinical interaction, participants underwent a brief review of the study protocol and procedures. Each participant was then fitted with eye-tracking glasses. For those with corrected vision who typically wear glasses, a diopter kit was employed to ensure proper vision while wearing the eye-tracking device. This involved swapping the default non-prescription lenses with appropriate corrective lenses. Following the setup, participants completed a 5-point calibration process to ensure accurate eye-tracking data collection. After completing the calibration, a researcher initiated the recordings for the participants’ eye movements, video, and audio data. The participants playing the roles were directed to an office where the session was held. Each session was timed to last 15 min. During this time, the non-participating individual was provided a designated waiting area. Upon completion of each session, the participating individuals were immediately asked to complete assessment measures related to the session.

After the conclusion of all three role-play scenarios, the researchers conducted a debriefing session. This allowed for a comprehensive explanation of the study’s objectives and provided participants with an opportunity to ask questions or voice any concerns.

### 2.3. Measures

Screening questionnaires and self-report measures were administered via Qualtrics, a secure online survey platform. The questionnaires included a demographics questionnaire, the Working Alliance Inventory—Short Form (WAI–S) [27,28,29,30,31], and the Session Rating Scale (SRS) [32].

Demographics Questionnaire. A demographics questionnaire including items on age, sex, gender, ethnicity, race, education, etc.

Working Alliance Inventory—Short Form. The WAI–S was used in this study to measure respondents’ perceptions of the client–counselor therapeutic alliance. The WAI–S is a 12-item, 7-point Likert-type (1 = never, 7 = always) version of the longer, 36-item WAI scale [30], with comparable psychometric properties reported for both versions [31]. The WAI–S includes three subscales: task agreement (e.g., “My therapist and I agree about the things I will need to do in therapy to help improve my situation”), goal agreement (e.g., “My therapist and I are working toward goals that we both agree on”), and bond (e.g., “My therapist and I trust one another”). The WAI is the most widely used self-report alliance scale and has shown strong reliability and validity [31]. WAI–S total scores range from 12 to 84, with higher scores indicating stronger perceived therapeutic alliance. Scales were slightly adapted for both therapist and client versions to assess agreement. Sum scores were used in all analyses. In the current study, the WAI-S showed good internal reliability for the client (α = 0.88) and acceptable reliability for the therapist (α = 0.75).

The Session Rating Scale (SRS). The SRS is a four-item visual analog scale designed to assess key dimensions of the therapeutic relationship. It is administered, scored, and discussed at the end of each session to elicit proximal feedback [32]. This manuscript focuses on the overall item: “Please rate the session Overall from 0 = There was something missing in the session today. 100 = Overall, today’s session was right for me.” Scales were slightly adapted for both clinician and therapist versions.

**Figure 1:**
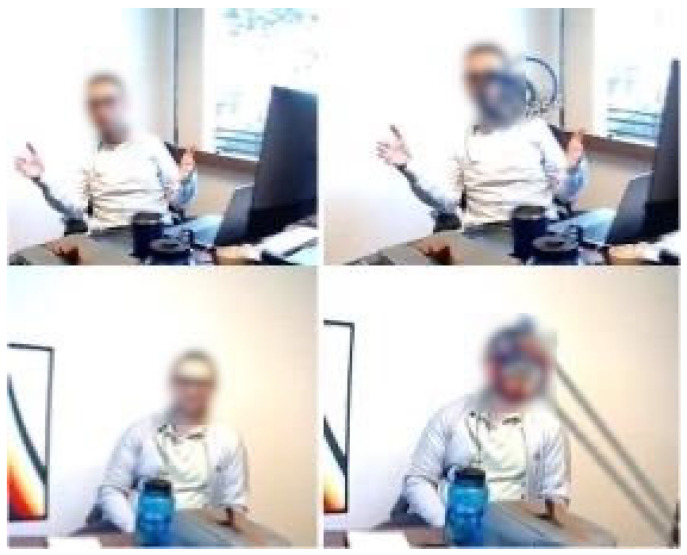
Authors MR and RH wearing the Neon eye tracking glasses, faces blurred to protect privacy. Images show world camera video recording (**left** panels) and fixation overlay (**right** panel).

Wearable Eye Trackers. Eye movement and audio data were collected using Pupil Labs Neon wearable eye-tracking glasses [33], which record gaze data at a sampling rate of 200 Hz. This high-frequency sampling allows for the precise detection of saccades, fixations, and smooth pursuit eye movements, making it suitable for detailed analyses of visual attention and gaze behavior in naturalistic environments [34]. In addition to capturing binocular eye position data, the Neon glasses include a forward-facing “world camera” that records high-resolution video of the participant’s field of view, along with synchronized ambient audio. This integration enables researchers to contextualize gaze behavior within dynamic real-world settings, such as conversations. The lightweight and unobtrusive design of the Pupil Labs Neon facilitates prolonged wear and supports ecological validity in studies involving mobile eye tracking (see Figure 1).

### 2.4. Data Analysis

Gaze data were extracted using Pupil Labs v7.4 [35] with the Face Mapper enrichment, which applies the RetinaFace algorithm to robustly detect faces in the scene video. For each frame, the system identifies facial bounding boxes and key landmarks (e.g., eyes, nose, mouth corners), enabling accurate mapping of gaze fixations onto facial regions. Audio data was extracted from the video stream and submitted first to a diarization process to separate audio between speakers (client and therapist) and then to a transcription process.

Cross Recurrence Quantification Analysis (CRQA). Gaze data (face vs. not face) and audio data (speaking vs. not speaking) for each dyad were entered into a multimodal cross recurrence quantification analysis using the crqa package in R version 4.5.1 [24]. We used the hamming distance method to determine recurrences, given our data was categorical. We extracted several key parameters: Recurrence Rate (RR): The proportion of recurrent points in the recurrence plot, reflecting overall synchrony between time series. Higher RR values indicate greater alignment in dyadic behavior; Number of Recurrent Lines (NRLINE): The total number of recurrent diagonal line structures, capturing the frequency of sustained interaction patterns. More recurrent lines suggest frequent and structured coordination; Mean Diagonal Line Length (L): The average length of diagonal lines, indicating the stability of coordinated patterns over time. Longer diagonal lines suggest prolonged periods of synchronization; Trapping Time (TT): The average length of vertical lines in the recurrence plot, measuring how long a system stays in the same state. Longer trapping times suggest that dyads maintain shared behavioral states for extended periods; Laminarity (LAM): The proportion of recurrent points forming vertical structures, representing periods of stationarity in the interaction. High LAM values indicate that dyadic behavior remains stable rather than continuously fluctuating.

Language Analysis of the Simulated Clinical Interactions. The transcripts of the interactions for each dyad were analyzed for the client and therapist separately based on each exchange with linguistic features averaged across the exchange, to be tested as predictors of working alliance. Lexical complexity was measured via Type-Token Ratio (TTR); sentiment analysis included both average emotional tone (AFINN) and variability in sentiment across turns. Turn-taking complexity was estimated by words per turn. Finally, syntactic complexity was evaluated using dependency parsing to capture sentence structure and word length as proxies for linguistic depth.

Bayesian Linear Regression. Bayesian linear regression was conducted in R using the brms package version 2.22 [36]. Independent variables were RR, NRLINE, L, TT, and LAM, extracted from CRQA as described above. Initially we tested additional parameters from CRQA, but examination of variance inflation factor (VIF) indicated that only inclusion of the parameters listed above yielded acceptable VIF (<5). We conducted exploratory analyses based on text analyses of the interactions to probe predictors of client working alliance. We focused on linguistic markers that were thought to be related to content and depth as highlighted above in the methods. Independent variables for the exploratory analyses were related to sentiment, lexical complexity, and linguistic depth as described above. Working Alliance Short Form (client and therapist versions) and the overall score from the session rating scale (for client and therapist). Additionally, we computed the difference between client and therapist ratings for each of the two measures to explore predictors of discrepancy in working alliance for our primary analyses.

We report how we determined our sample size, all data exclusions, all manipulations, and all measures in the study. The study was not pre-registered. A total of 15 min of eye tracking and audio data were collected for each participant in each dyad, these data are not available to be shared given their inherently identifiable nature. All de-identified data and R syntax used for results reported in the manuscript are available to be shared on request to the corresponding author.

## 3. Results

Participants in dyads played a role as either the therapist, or client during simulated clinical interactions lasting 15 min. On average, therapists spoke for 41% of the interaction and their gaze was on the face of the client an average of 57% of the time while listening and 56% while speaking. Clients spoke for an average of 42% of the interaction and fixated 41% of the time on the face of the therapist while listening and 44% while speaking.

Overall, those in the role of clients rated the therapeutic alliance and quality of the interaction highly for all the measures (M = 63.17, SD = 7.17). Similarly, those in the role of therapists rated the alliance highly (M = 60.07, SD = 6.68). Within session correlation between alliance measures was strong r(25) = 0.68, 95% confidence interval (CI) [0.40, 0.84]. Overall session ratings were also high for the client (M = 91.71, SD = 16.04) and the therapist (M = 83.78, SD = 16.23) with a very strong correlation: r(25) = 0.80, 95% CI [0.61, 0.91].

**Do the Dynamics of the Interactions Play a Role in Therapeutic Alliance?**

Using Multidimensional Cross Recurrence Quantification Analysis the parameters described above were tested in a series of Bayesian regression models, described fully in Table 2.

No meaningful predictors were identified for client rated working alliance, with an overall model R2 = 0.20; for therapist rated working alliance, TT positively associated b = 4.62, 95% CI [0.07, 8.94] and LAM negatively associated b = −4.77, 95% CI [−8.46, −1.01], with an overall model R2 = 0.42. Estimating the discrepancy for each dyad in working alliance in a separate model, we found that NRLINE predicted the difference between client and therapist working alliance b = −4.29, 95% CI [−8.12, −0.38]. There were no associations between CRQA parameters and overall client (R2 = 0.23) or therapist session ratings (R2 = 0.22); however, NRLINE was associated with the difference in SRS ratings between client and therapist, b = −6.67, 95% CI [−12.89, −0.25].

**Does the Language Use of the Interactions Play a Role in Therapeutic Alliance?**

For client language markers, dependency complexity b = 4.26, 95% CI [2.23, 6.29], words per turn b = −2.92, 95% CI [−5.37, −0.54], and sentiment variability b = 2.41, 95% CI [0.05, 4.78] predicted client working alliance, (R2 = 0.36). There were no associations between language markers and overall client (R2 = 0.15) session ratings.

## 4. Discussion

These preliminary findings partially supported our hypothesis that therapeutic alliance in clinical training interactions is associated with certain behaviors consistent with stronger interpersonal engagement. Overall, the mock sessions were interpreted to have gone well and interestingly, both gaze and speech were relatively balanced between therapist and client. It is especially notable that gaze on face was high when speaking. This may indicate a degree of deliberate engagement in the interaction that is less typical in general social situations where gaze tends to be greater when listening rather than speaking [37]. However, there is limited prior evidence from eye tracking of dyadic gaze behaviors during clinical interactions to qualify this finding more explicitly.

CRQA parameters identified were specifically related to the therapist rated working alliance, but not the client rated working alliance. Specifically, the therapist regression findings based on the TT parameter suggested that a more dynamic, interactive exchange (rather than a monotonous or rigid conversation) may have led therapists to perceive a stronger alliance. High values of LAM on the other hand indicate repetitive, sustained patterns in the interaction. This finding may indicate that when the interaction becomes too repetitive or stuck in a particular conversational state, therapists perceive the alliance as weaker. One possible explanation for the findings on the therapist WAI but not the client WAI is that the therapist was likely more aware of the dynamics of the mock-session. Those in the therapist role might have been more conscious of how the session was going in terms of how dynamic the interaction was and the flow of the conversation. In contrast, those in the client role might have focused less on the experience of the relationship in terms of those patterns. The client’s perception of the alliance might have been more influenced by subjective factors such as emotional support, comfort, and connection rather than predictability or stability of the interaction over time. We did identify the difference between the WAI scale for therapist and client as linked to NRLINE which represents how structured the interactions were: meaning that clients rated the alliance lower than therapists as NRLINE increased. An interpretation of this finding related to NRLINE could be that those in the client role found highly structured interactions as less personal or authentic, while therapists might associate more structure of the session as reflecting better progress. Thus, perceptions of predictability in therapy interactions may create a mismatch between therapist and client alliance ratings.

Exploratory findings highlighted the importance of linguistic complexity, sentiment (affective content of what was discussed), and depth of the interactions in enhancing the clients’ ratings of working alliance. Whereas CRQA findings were only related to therapist rated working alliance, linguistic findings showed that clients rated higher working alliance associated with complexity, notably this included depth and variability in sentiment. This finding underscores a key distinction in how clients and therapists may perceive the therapeutic relationship. Clients appear to weigh affective, content, and emotional depth as central to their working alliance with the therapist. In contrast, therapists’ ratings of the alliance may be more closely tied to the structural dynamics of the interaction, such as the flow and synchrony captured by CRQA findings.

### Limitations

There were several important limitations to discuss. First, the sample size was small. This is especially important in the context of the relatively high ratings for both measures of alliance used—the lack of variability means that it may be harder to detect effects. Second, these were brief mock-sessions (lasting only 15 min), not true therapy sessions. This limits the potential of generalizability of findings to real clinical interactions and may have also played a role in the high ratings of working alliance. Additionally, the majority of participants were PhD students in clinical psychology which represents a different demographic than many Master’s level clinical training programs. We investigated the working alliance from several self-report measures and characterizing the session in other ways (e.g., qualitatively) may have utility for better characterizing how interaction dynamics and language use play a role in working alliance in clinical settings. Lastly, it is possible that wearing eye trackers influences gaze to some degree. There is no evidence to suggest this, but it is a possibility we cannot discount.

## 5. Conclusions

Taken together, our findings build on prior work emphasizing the value of multimodal data in examining working alliances within clinical contexts. Our central hypothesis that our findings support the hypothesis that the therapeutic alliance in clinical training interactions is associated with behavioral patterns indicative of stronger interpersonal engagement was partially supported. By integrating gaze, speech, and linguistic data, we leveraged a computational framework capable of capturing the complexity of interpersonal dynamics during therapy. This approach enables a richer understanding of alliance-related processes than traditional self-report or unimodal methods. Future research should consider expanding the range of input modalities and applying time-sensitive analytic techniques to model the evolution of alliance over the course of longer clinical interactions. Notably, developing an automated feedback system that delivers real-time insights to clinicians would represent a meaningful extension of this work. The methods introduced here have potential applicability across a range of settings where relational processes are critical, including a wide range mental health settings, and also in physician training programs (i.e., medical school and/or residency) and in the development of better human–computer interactions.

## Figures and Tables

**Table 1:** Demographics.

	M	SD
Age	30.56	7.88
	%	N
Sex (Female)	74	20
Hispanic/Latinx	26	7
Race		
Asian	19	5
Hispanic/Latinx	15	4
Native Hawaiian or Other Pacific Islander	4	1
White	44	12
More than 1 Race	15	4
Not Otherwise Specified: (Middle Eastern, Not White)	4	1
Program		
PhD	85	23
PsyD	7	2
MA Counseling	7	2
First Generation Student	26	7

Note: % may not sum to 100 due to rounding.

**Table 2:** Regression Coefficients for Primary Analyses Using Multimodal Cross Recurrence Quantification Analysis.

Variable	WAI-Client	WAI-Therapist	SRS-Client	SRS-Therapist
	b [95% confidence interval]			
Intercept	63.18 [60.43, 65.94]	60.04 [57.86, 62.20]	92.87 [87.64, 97.96]	83.99 [77.86, 90.27]
RR	0.94 [−4.73, 6.69]	−0.74 [−5.45, 4.02]	1.07 [−8.74, 10.68]	1.62 [−8.42, 11.75]
NRLINE	−1.38 [−6.64, 3.80]	2.20 [−2.21, 6.54]	−3.00 [−12.09, 6.00]	1.94 [−7.68, 11.35]
L	1.84 [−2.71, 6.40]	1.22 [−2.32, 4.90]	3.07 [−5.44, 11.63]	0.48 [−8.31, 9.47]
TT	1.85 [−3.51, 7.12]	4.62 [0.07, 8.94]	3.42 [−5.93, 12.68]	4.87 [−4.64, 14.29]
LAM	−2.88 [−7.33, 1.67]	−4.77 [−8.46, −1.01]	0.70 [−7.44, 8.78]	−1.65 [−9.93, 6.86]

Note. RR = Recurrence Rate; NRLINE = Number of Recurrent Lines; L = Mean Diagonal Line Length; TT = Trapping Time; LAM = Laminarity. Coefficients are partially standardized (z-scored for the independent variables).

## Data Availability

The raw de-identified data supporting the conclusions of this article will be made available by the authors on request.
